# Ab externo implantation of the MicroShunt, a poly (styrene-*block*-isobutylene-*block*-styrene) surgical device for the treatment of primary open-angle glaucoma: a review

**DOI:** 10.1186/s40662-019-0162-1

**Published:** 2019-11-15

**Authors:** Omar Sadruddin, Leonard Pinchuk, Raymund Angeles, Paul Palmberg

**Affiliations:** 1 Santen Inc, Emeryville, CA USA; 2 InnFocus Inc., a Santen Company, Miami, FL USA; 3 Bascom Palmer Eye Institute, University of Miami Miller School of Medicine, Miami, FL USA

**Keywords:** Ab externo, Glaucoma, Micro-invasive glaucoma surgery, MicroShunt, Mitomycin C, SIBS polymer

## Abstract

Trabeculectomy remains the ‘gold standard’ intraocular pressure (IOP)-lowering procedure for moderate-to-severe glaucoma; however, this approach is associated with the need for substantial post-operative management. Micro-invasive glaucoma surgery (MIGS) procedures aim to reduce the need for intra- and post-operative management and provide a less invasive means of lowering IOP. Generally, MIGS procedures are associated with only modest reductions in IOP and are targeted at patients with mild-to-moderate glaucoma, highlighting an unmet need for a less invasive treatment of advanced and refractory glaucoma. The PRESERFLO® MicroShunt (formerly known as InnFocus MicroShunt) is an 8.5 mm-long (outer diameter 350 μm; internal lumen diameter 70 μm) glaucoma drainage device made from a highly biocompatible, bioinert material called poly (styrene-*block*-isobutylene-*block*-styrene), or SIBS. The lumen size is sufficiently small that at normal aqueous flow hypotony is avoided, but large enough to avoid being blocked by sloughed cells or pigment. The MicroShunt achieves the desired pressure range in the eye by draining aqueous humor from the anterior chamber to a bleb formed under the conjunctiva and Tenon’s capsule. The device is implanted ab externo with intraoperative Mitomycin C via a minimally invasive (relative to incisional surgery) surgical procedure, enabling precise control of placement without the need for gonioscopy, suture tension control, or suture lysis. The implantation procedure can be performed in combination with cataract surgery or as a standalone procedure. The MicroShunt received Conformité Européenne (CE) marking in 2012 and is intended for the reduction of IOP in eyes of patients with primary open-angle glaucoma in which IOP remains uncontrolled while on maximum tolerated medical therapy and/or in which glaucoma progression warrants surgery. Three clinical studies assessing the long-term safety and efficacy of the MicroShunt have been completed; a Phase 3 multicenter, randomized clinical study comparing the MicroShunt to primary trabeculectomy is underway. In preliminary studies, the MicroShunt effectively reduced IOP and use of glaucoma medications up to 3 years after implantation, with an acceptable safety profile. This article summarizes current literature on the unique properties of the MicroShunt, the preliminary efficacy and safety findings, and discusses its potential use as an alternative to trabeculectomy for glaucoma surgery.

## Background

Trabeculectomy and tube shunt surgery remain the most commonly performed incisional intraocular pressure (IOP)-lowering glaucoma procedures for the treatment of moderate-to-severe and refractory glaucoma [1]. These surgical methods help to address the suboptimal adherence associated with pharmacologic therapies [2]. However, despite being efficacious at lowering IOP, incisional surgery techniques are associated with a requirement for substantial post-operative management [1, 3].

Micro-invasive glaucoma surgery (MIGS), or minimally invasive glaucoma surgery, is a term used to describe an increasingly available group of surgical procedures [4]. MIGS procedures aim to reduce intra- and post-operative management and offer a less invasive means of reducing IOP than traditional glaucoma surgery, with the goal of reducing dependency on topical medications [2, 5]. Reduction of IOP by MIGS is achieved by either increasing trabecular outflow by bypassing the trabecular meshwork, increasing uveoscleral outflow via suprachoroidal pathways, reducing aqueous production from the ciliary body, or creating a subconjunctival drainage pathway for aqueous humor [5]. Although MIGS procedures benefit from an improved safety profile compared with traditional surgery, differences in terms of outflow pathway, ab interno versus ab externo approach, and whether a bleb is created lead to variations in target patient population, efficacy, and device- or procedure-related adverse events (AEs) [4–6]. Most MIGS procedures developed to date have been associated with only modest reductions in IOP and are therefore targeted at patients with mild-to-moderate glaucoma, highlighting an unmet need for minimally invasive treatment of moderate-to-severe and refractory glaucoma [5].

The invention of a novel synthetic, thermoplastic, elastomeric biomaterial (poly [styrene-*block*-isobutylene-*block*-styrene]; SIBS) that resists biodegradation in the body, paired with the need for a safe and effective method for treating glaucoma, resulted in the development of a SIBS-based glaucoma drainage device known as the PRESERFLO® MicroShunt (formerly known as the InnFocus MicroShunt) [7, 8]. The MicroShunt is a subconjunctival glaucoma drainage device that facilitates aqueous humor outflow to a bleb, providing substantial IOP reductions [9]. The MicroShunt received Conformité Européenne (CE) marking on January 9, 2012 in Europe [7], and a US Investigational Device Exemption (IDE) to initiate a Phase 3 clinical study was granted by the US Food and Drug Administration (FDA) in May 2013. To date, three clinical studies assessing the long-term safety and efficacy of the MicroShunt have been completed [10–12], and a multicenter clinical study comparing the MicroShunt to primary trabeculectomy is currently underway [7, 13].

This review will present a detailed overview of the development, material and design, surgical procedure, key published data from completed studies, and future perspectives on the MicroShunt.

## Main text

### Development of the MicroShunt

**Fig. 1:**
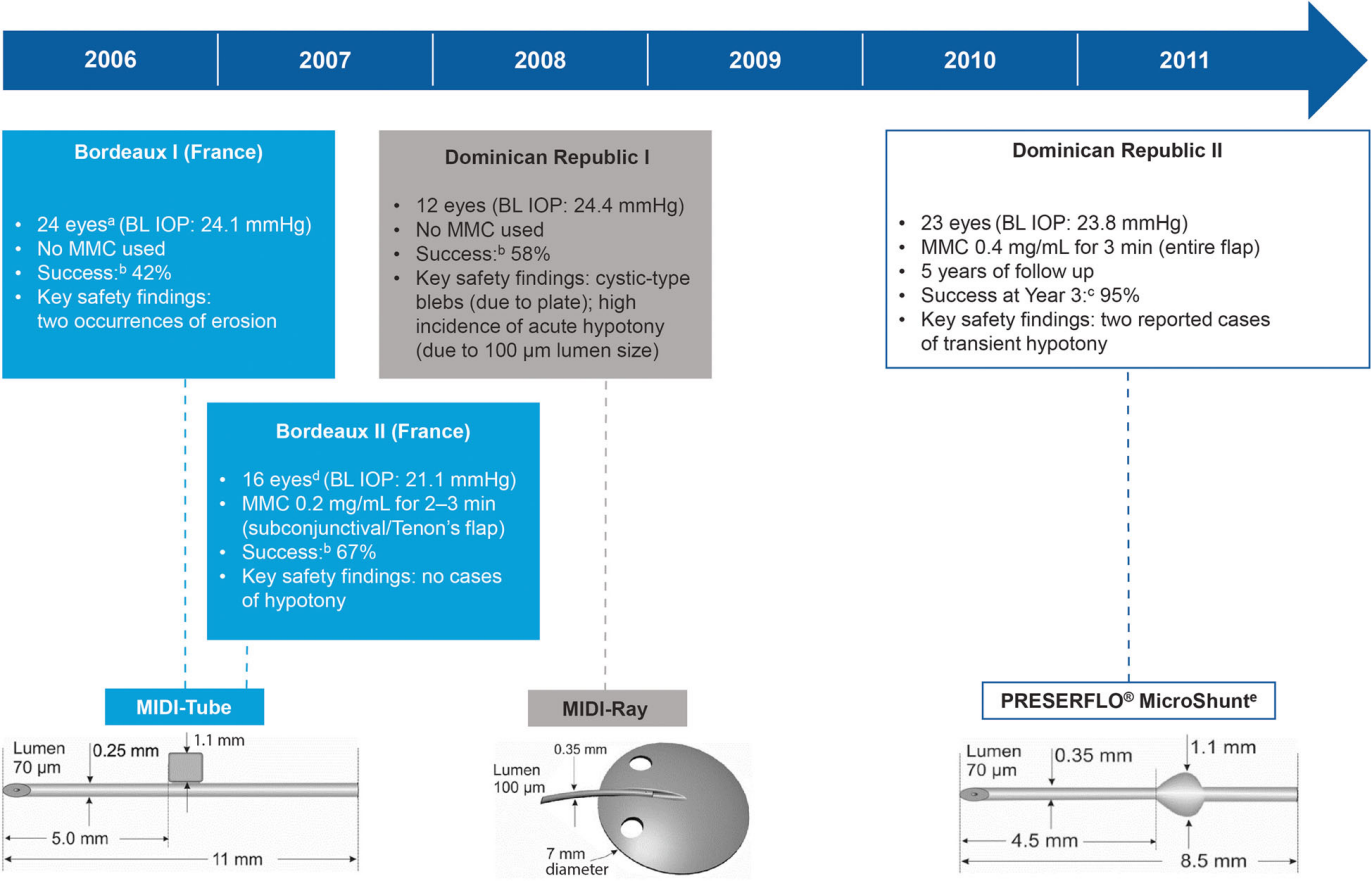
Human pilot feasibility studies of three iterations of a SIBS-based glaucoma drainage microtube [7–9]. ^a^Advanced glaucoma cases, with about half of the eyes failing previous trabeculectomy. ^b^Qualified success was defined as IOP ≤ 21 mmHg with a ≥ 20% reduction in IOP from baseline, with or without glaucoma medication and with no further incisional procedure. ^c^Qualified success was defined as IOP ≤ 14 mmHg with a ≥ 20% reduction in IOP from baseline, with or without glaucoma medication and with no reoperation for glaucoma. ^d^Eleven of these patients had failed previous incisional procedures. ^e^Previously known as MIDI-Arrow and InnFocus MicroShunt. *BL* = baseline; *IOP* = intraocular pressure; *MIDI* = Miami InnFocus drainage implant; *MMC* = Mitomycin C; *SIBS =* poly (styrene-*block*-isobutylene-*block*-styrene). (Reprinted from Pinchuk L, et al. J Biomed Mater Res Part B 2017;105B:211–21. Copyright© 2016 Society for Biomaterials. Published with permission of John Wiley & Sons, Ltd.[8].)

The development of SIBS and subsequently the MicroShunt was an iterative process that occurred over the course of 20 years [8, 14]. Three major iterations of MicroShunt design were investigated before arriving at the current design (Fig. 1) [7, 8].

The first two design iterations were initially investigated in both acute and chronic rabbit eye biocompatibility studies [8]. The Miami InnFocus Drainage Implant (MIDI)-Tube (an 11 mm SIBS tube with a 1 mm SIBS tab) was assessed in two studies at the Bascom Palmer Eye Institute Ophthalmic Biophysics Center (OBC) (Miami, FL, USA) [8] and then confirmed in a good laboratory practice (GLP) study conducted at the North American Science Associates contract facility (Northwood, OH, USA) [8, 15]. Following this, the MIDI-Ray (a 350 μm diameter SIBS tube with a 100 μm lumen and a 7 mm diameter SIBS plate) was investigated in a chronic, non-GLP animal study conducted at the Bascom Palmer Eye Institute OBC [8]. Based on positive results from the biocompatibility studies, the SIBS-based devices then underwent clinical testing [8].

Four human pilot feasibility studies (Bordeaux I and II, and Dominican Republic I and II) were conducted over a 4-year period to establish the optimal design, best implantation techniques, and requirement for Mitomycin C (MMC) (Fig. 1) [8]. Promising results from the Dominican Republic II study with the MicroShunt (qualified success of 95% at 3 years and only two reported cases of transient hypotony) resulted in the decision to proceed with the MicroShunt design with MMC in further clinical evaluations [8, 9].

### Material and design of the MicroShunt

**Fig. 2:**
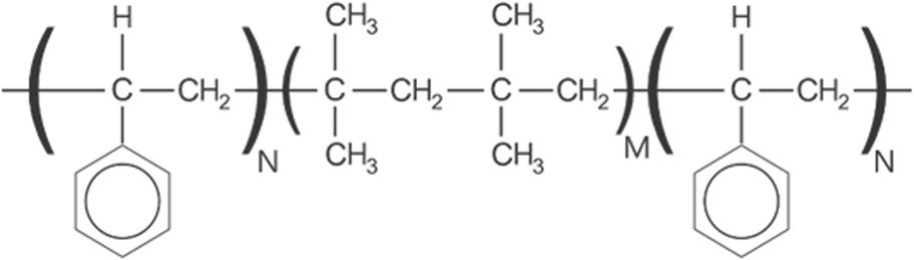
Simplified chemical structure of SIBS Where M > > N. *M* = number of isobutylene units; *N* = number of styrene units; *SIBS* = poly (styrene-*block*-isobutylene-*block*-styrene).

The MicroShunt is made from SIBS (Fig. 2) [7], which is synthesized by a living cationic polymerization technique [8, 16]. The inert, soft, and flexible thermoformable elastomeric properties of SIBS enable the MicroShunt to conform to the curvature of the eye [17].

In preclinical studies, SIBS demonstrated biostability in the eye, with a lack of biodegradation byproducts, resulting in reduced chronic inflammation and minimal scar formation [17]. In 2003, Dr. Jean-Marie Parel and his team at the Bascom Palmer Eye Institute OBC laboratory conducted a study comparing the effects of silicone tubes versus SIBS implants in the corneal stroma and sub-Tenon’s space of New Zealand White rabbit eyes [8, 17]. SIBS implants were found to be biocompatible in the rabbit model and maintained 100% flow patency at 6 months [17]. Results showed reduced collagen deposition in the SIBS group compared with the silicone group; furthermore, myofibroblasts were not observed in tissue surrounding the SIBS implants, whereas silicone implants were shown to induce expression of cellular components responsible for scarring [17].

These positive preclinical findings demonstrating SIBS biocompatibility in ophthalmology are in line with real-world experience with SIBS in cardiology. The SIBS-coated TAXUS® (Boston Scientific Corporation, Natick, MA, USA) is a cardiac stent that releases the antiproliferative drug paclitaxel in the coronary artery as a means of minimizing restenosis [18]. TAXUS® has been implanted in more than a million patients worldwide, with a well-established safety profile [8, 18]. In vitro and in vivo studies of the TAXUS® cardiac stent have confirmed no biodegradation and minimal inflammation, highlighting the versatility of SIBS as a biocompatible polymer [19].

**Fig. 3:**
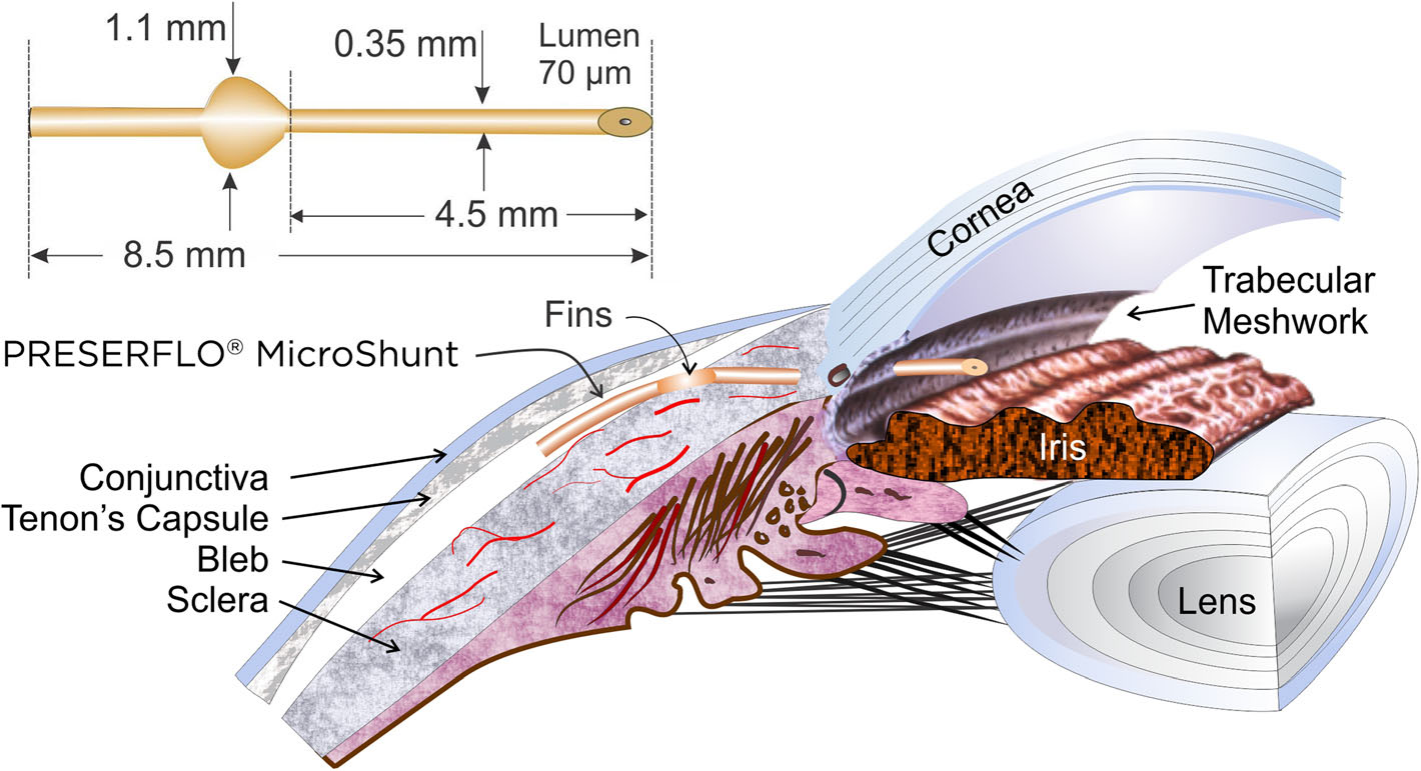
Dimensions of the MicroShunt and placement in the eye. (Adapted from Pinchuk L, et al. Regen Biomater. 2016;3:137–42 [7].)

**Fig. 4:**
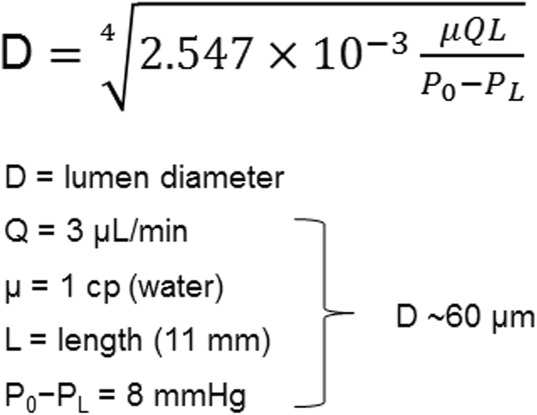
Hagen-Poiseuille equation for laminar flow 60 μm is an example using the variables listed.

The MicroShunt is an 8.5 mm-long (350 μm outer diameter; 70 μm lumen) surgical device that has been designed for implantation in glaucomatous eyes to achieve the desired pressure range by draining the aqueous humor from the anterior chamber through the sclera to under the conjunctiva and Tenon’s capsule to form a bleb (Fig. 3) [7]. The length of the device was designed to allow it to be positioned through a 3 mm-long scleral needle tunnel with the outflow end above the scleral surface behind the excursion of the upper eyelid [9]. The lumen size was approximated using the Hagen-Poiseuille equation for laminar flow (Fig. 4) [7] and was optimized in a rabbit eye implant study [20]. The rabbit eye implant study conducted by Arrieta et al. investigated SIBS implants with differing internal lumen diameters (70, 100, and 150 μm) in New Zealand White rabbit eyes and concluded that 70 μm and 100 μm SIBS implants resulted in fewer post-operative complications compared with the 150 μm implant [20]. The study also concluded that a lumen diameter of 70 μm was found to be adequate to prevent chronic hypotony [20], while also being sufficiently large to prevent clogging (the 70 μm diameter of the lumen is larger than the 40–50 μm diameter of a sloughed endothelial cell) [7].

Located halfway down the MicroShunt is a 1.1 mm wingspan fin (Fig. 3) that sits within a shallow pocket in the sclera [7, 9]. The fin prevents migration of the device into the eye [7], holds the device in the pocket, preventing any peri-annular leakage [9], and orients the device into the correct position, with the bevel facing the cornea, to enable clearance of debris if the lumen entrance becomes blocked [7].

### Surgical implantation of the MicroShunt

The MicroShunt drains aqueous humor from the anterior chamber to a bleb formed under the conjunctiva and Tenon’s capsule [7]. The subconjunctival fluid collected within the bleb is resorbed either directly into the episcleral venous system [7], into the tear film via microcysts (naturally occurring channels in the conjunctiva) [7, 21], or via orbital lymphatics [22, 23]. Drainage of aqueous humor through this route by the MicroShunt bypasses high resistance in the trabecular meshwork, as well as Schlemm’s canal, the collector channels, and the scleral venous plexus [7, 8].

**Fig. 5:**
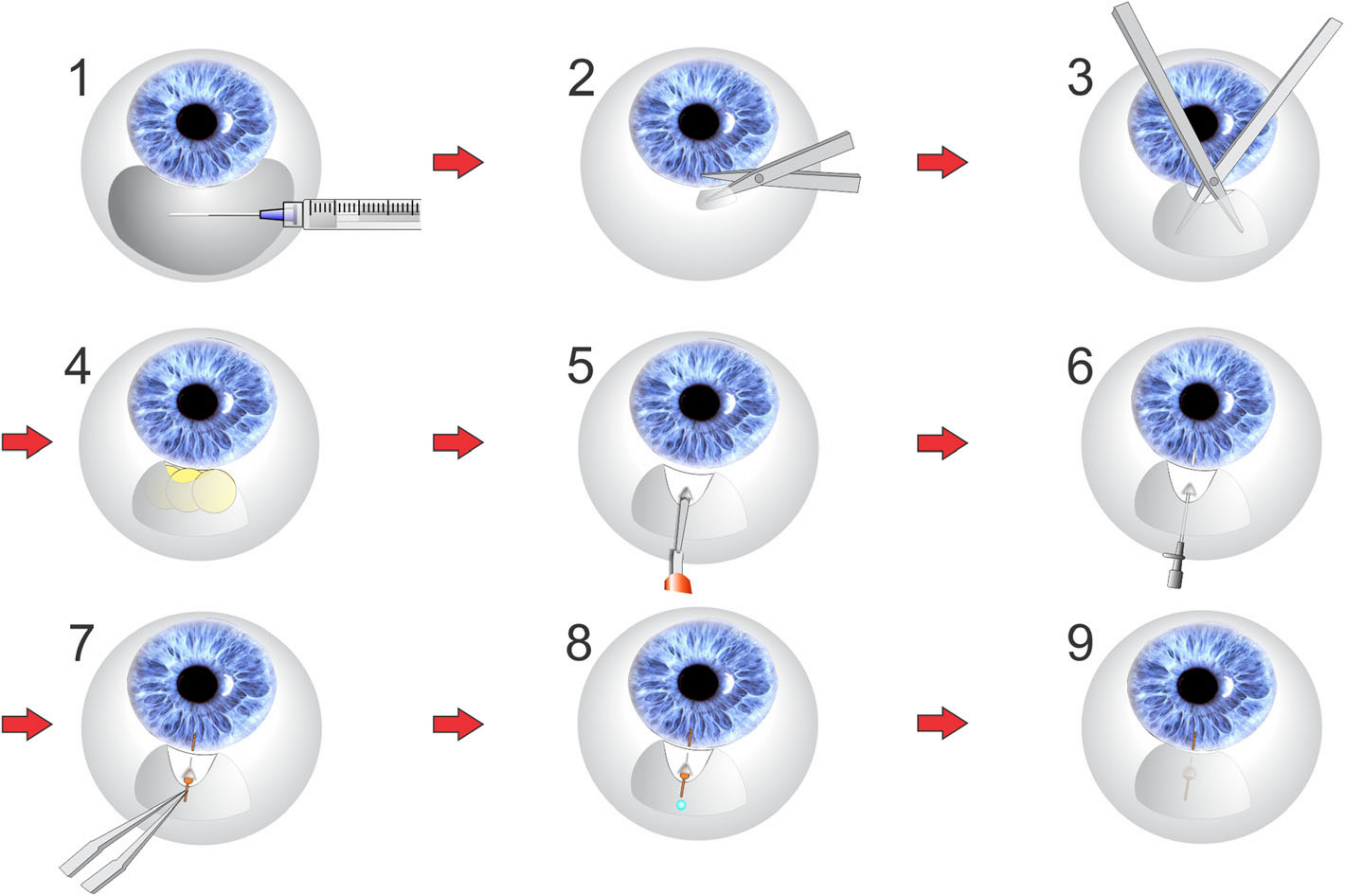
Surgical procedure for MicroShunt implantation. (Adapted from Pinchuk L, et al. Regen Biomater. 2016;3:137–42 [7].) 1. Anesthetic is administered beneath the conjunctiva (anesthetic can be injected locally or as a peribulbar block, or applied topically); 2. An incision is made parallel to the limbus and under Tenon’s capsule; 3. Blunt scissors are used to dissect the Tenon’s from the sclera over one to two quadrants and deep to the equator; 4. Following hemostasis using bipolar diathermy (not shown), MMC-soaked sponges are placed in the pocket, which is then rinsed with sterile saline solution; 5. A 1 mm-wide, 1–2 mm-long shallow scleral pocket is made 3 mm posterior to the limbus; 6. A needle is passed through the scleral pocket into the anterior chamber, approximately bisecting the cornea and iris at the level of the trabecular meshwork;^a^ 7. The MicroShunt is threaded through the pocket and needle tunnel with forceps, and the fins of the device are wedged into the scleral pocket; 8. The flow of aqueous humor from the anterior chamber to the flap is confirmed by drop observation; 9. The distal end of the device is tucked beneath the conjunctiva and Tenon’s capsule which are then pulled over the MicroShunt and sutured back to the limbus with 10–0 nylon sutures ^a^This step is consistent with the EU labeling; in the USA, a double-step knife is used instead of a needle to create the tunnel into the anterior chamber [13]. *MMC* = Mitomycin C.

The surgical procedure for the MicroShunt (illustrated in Fig. 5) is minimally invasive (relative to trabeculectomy), and the device is implanted via an ab externo approach [7, 9, 24]*.*

A fornix-based subconjunctival and sub-Tenon’s flap is dissected at the nasal or temporal quadrant over a circumference of 90 to 120 degrees, to at least 8 to 10 mm posterior to the limbus. Following placement of MMC-soaked sponges in the flap for 2 to 3 min of exposure, a 3 mm marker is used to mark a point 3 mm from the middle border of the surgical limbus in the blue-gray zone. At the distally-marked point on the sclera, a 1 mm width knife is used to incise a shallow triangular pocket in the sclera (large enough to seat the fins of the MicroShunt). A needle is then used to create a transscleral tunnel from the apex of the scleral pocket into the anterior chamber. Using forceps, the MicroShunt is threaded, bevel up and fins flat, into the transscleral tunnel. The fins are then wedged into the scleral pocket. It is important that flow through the MicroShunt is checked prior to closure of Tenon’s capsule and the conjunctiva. Flow is confirmed visually by first observing a percolation of air and aqueous humor from the distal end of the device. Once air is purged from the tube, a drop of aqueous humor will slowly grow on the distal end of the device. As the volume of the drop increases, flow could be erroneously perceived as decreasing; however, volume increases to the third power of flow and so flow is difficult to judge when the drop is too large. It is prudent to wipe the drop away at times with a sponge and visualize a small drop to confirm flow. The IOP should then be estimated at equilibrium flow to be about 6 mmHg or less, which can be effectuated by depressing the central cornea with a 30-G cannula. If flow through the lumen is not observed, then the following troubleshooting procedures can be performed: 1) ensure that the entrance to the MicroShunt is free of debris and not lodged in the iris or cornea; 2) increase IOP by injecting BSS through a paracentesis in the clear cornea; 3) use a 30-G cannula and inject BSS through the lumen of the MicroShunt to discharge air and prime the device; 4) check for fluid flow around the device as, if the fins are not seated correctly, the path of least fluid resistance can be around the MicroShunt instead of through the lumen of the device; 5) withdraw the MicroShunt slightly in the event that the fins are wedged too tightly in the pocket thereby constricting the lumen and preventing flow; 6) remove the MicroShunt and place in a new needle tunnel; 7) if none of these procedures initiate flow, remove the device and replace with a new MicroShunt. Following confirmation of flow, the distal end of the MicroShunt is tucked underneath Tenon’s capsule and the conjunctiva, ensuring that it is straight and free of tissue; sutures are then used to reposition Tenon’s capsule and the conjunctiva over the device and to the limbus [7, 9, 13].

It is of note that implantation of the MicroShunt can be performed in combination with cataract surgery, or as a standalone procedure [9]; furthermore, the implantation procedure does not require intraoperative gonioscopy, sclerostomy, or iridectomy [8, 25].

Traditional glaucoma-filtering surgeries routinely use MMC, and a Cochrane review confirmed that MMC is able to reduce the risk of failure in trabeculectomy [26]. This agent inhibits the proliferation of cells that form scar tissue [26, 27]; adjunctive application of MMC after filtering surgery, with or without needling, is performed to attenuate post-operative subconjunctival fibroblast proliferation and suppress excessive bleb scarring [27]. Intraoperative use of MMC has also been shown to reduce the risk of surgical failure and increase the surgical success rate in minimally invasive devices, including the MicroShunt [8]. Various MMC concentrations and application times have been used in glaucoma filtration surgery; a dose-response relationship, although observed in some studies, has not always been reported [28]. Concentration and application time of MMC during implantation of the MicroShunt vary in the literature; concentrations of 0.2–0.4 mg/mL and application times of 2–3 min have been reported [7–9].

### Clinical evaluation of the MicroShunt

Three clinical studies have been completed, and a further study is ongoing, assessing the long-term safety and efficacy of the MicroShunt (Table 1) [10–13].

**Table 1:** Complete and ongoing MicroShunt clinical studies

Study	Clinical study of the safety and performance of the Miami InnFocus Drainage Implant to relieve glaucoma symptoms [12]	Safety and performance of the Miami InnFocus Drainage Implant (MIDI Arrow) glaucoma drainage implant [11]	Postmarket study of the InnFocus MicroShunt [10]	InnFocus MicroShunt Versus Trabeculectomy Study (IMS) [13]
NCT number (other study ID)	NCT00772330 (INN003)	NCT01563237 (INN004)	NCT02177123 (INN007)	NCT01881425 (INN005)
Phase	1	2	Post market	3
Control	Single arm	Single arm	Single arm	Randomized, parallel assignment, single masked
Center(s)	1 (Dominican Republic)	1 (France)	6 (France, the Netherlands, Spain, Switzerland)	29 (France, Italy, the Netherlands, Spain, the UK, the USA)
Follow up (years)	5	2	2	2
Enrolled patients	23	72	100	889 (estimated)
Key inclusion criteria	Age: 18–85 years IOP: ≥ 18 and ≤ 40 mmHg inadequately controlled on tolerated medical therapy		Age: 18–85 years IOP: ≥ 18 and ≤ 35 mmHg on maximum tolerated medical therapy and/or where glaucoma progression warrants surgery	Age: 40–85 years IOP: ≥ 15 and ≤ 40 mmHg on maximum tolerated medical therapy
Key exclusion criteria	Need for glaucoma surgery combined with other ocular procedures other than cataract surgery or anticipated need for additional ocular surgery during the study		Previous incisional ophthalmic surgery (excluding uncomplicated cataract surgery), or argon laser, selective laser, or micropulse trabeculoplasty within 90 days of enrollment	Previous conjunctival incisional ophthalmic surgery, anticipated need for additional ocular surgery during the study
Primary outcome	IOP reduction relative to baseline at 12 months			> 20% IOP reduction from baseline at 12 months without increasing the number of glaucoma medications
Study start	October 2007	June 2011	April 2014	August 2013
Primary completion	November 2016	December 2016	November 2017	November 2018
Completion	January 2017	January 2017	November 2017	November 2019 (estimated)

*IOP* = intraocular pressure

### Key data from a MicroShunt clinical study

In a prospective, single-arm study conducted in the Dominican Republic (NCT00772330), the MicroShunt was implanted with MMC (0.4 mg/mL for 3 min) in 23 patients with primary open-angle glaucoma [9]. Of these patients, 14 received the MicroShunt alone, and 9 received the MicroShunt in combination with cataract surgery [9]. Three-year outcomes of the study are summarized in Table 2.

**Table 2:** Summary of key MicroShunt efficacy outcomes at Years 1–3 [9]

	Baseline	Year 1	Year 2	Year 3
Mean medicated IOP, mmHg ± SD	23.8 ± 5.3	10.7 ± 2.8	11.9 ± 3.7	10.7 ± 3.5
Mean number of glaucoma medications per patient ± SD	2.4 ± 0.9	0.3 ± 0.8	0.4 ± 1.0	0.7 ± 1.1
Qualified success,^a^ %	–	100	91	95

^a^Qualified success was defined as IOP ≤ 14 mmHg with a ≥ 20% reduction in IOP from baseline, with or without glaucoma medication and with no reoperation for glaucoma

*IOP* = intraocular pressure; *SD* = standard deviation

**Fig. 6:**
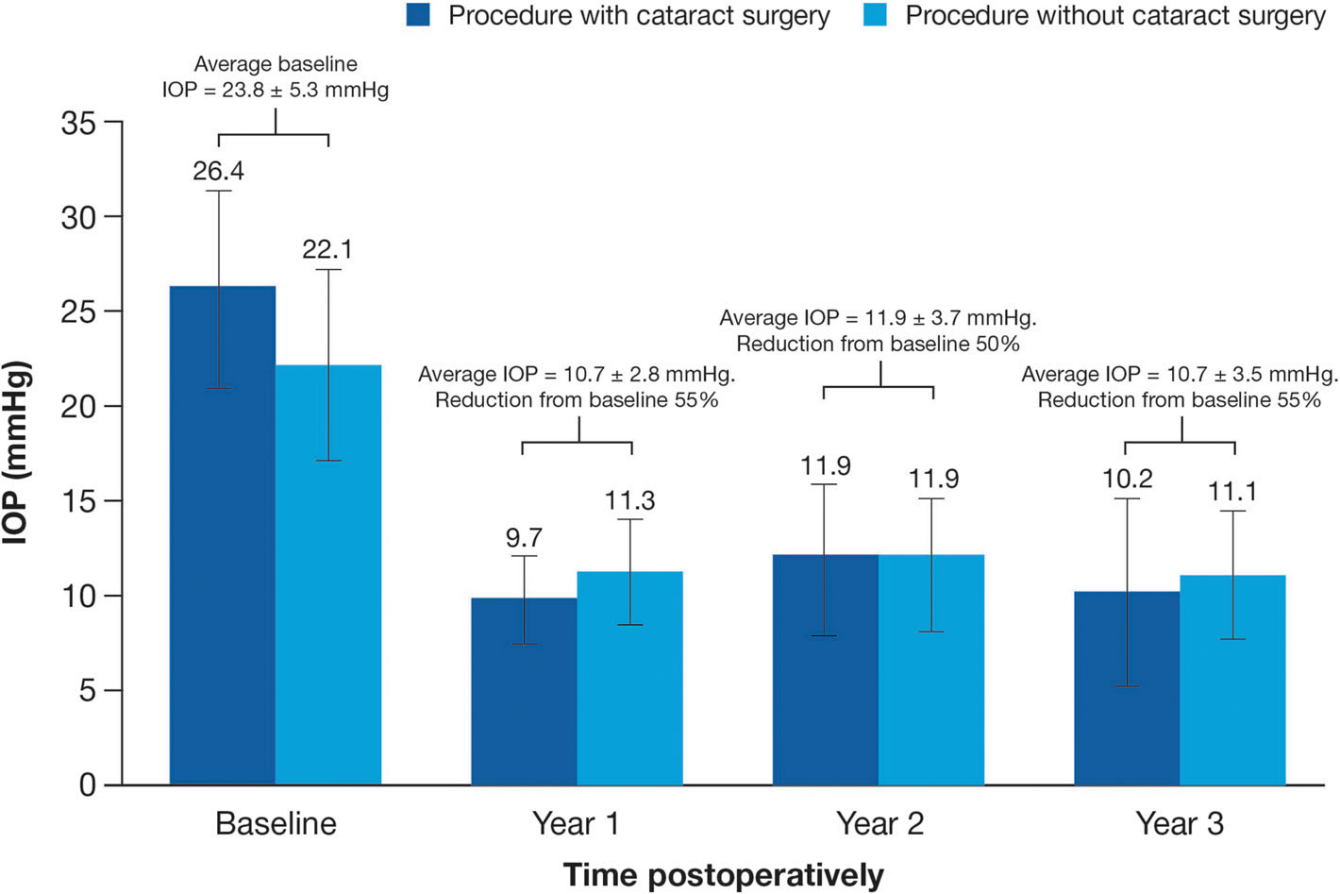
Mean medicated IOP levels from baseline up to Year 3. (Adapted from Pinchuk L, et al. Regen Biomater. 2016;3:137–42 [7].) *n* = 9 from baseline to Years 1, 2, and 3 for MicroShunt procedure with cataract surgery; *n* = 14, 13, and 13 from baseline to Years 1, 2, and 3, respectively for MicroShunt procedure without cataract surgery. *IOP* = intraocular pressure.

A similar IOP reduction was observed in patients who received the MicroShunt alone and in patients who received the MicroShunt in combination with cataract surgery (Fig. 6) [9]. At the Year 2 visit, there were two eyes that did not demonstrate an IOP of ≤14 mmHg and ≥ 20% reduction in IOP from baseline (having an IOP of 18 and 19 mmHg, respectively); at Year 3, only one eye did not meet the criteria for qualified success (with an IOP of 16 mmHg) [9]. One eye required reoperation [9]. At Year 3, the overall mean reduction in glaucoma medications was 71%, with 64% of patients no longer taking IOP-lowering medication [9].

Twenty-one post-operative AEs were noted in seven patients. Two of the seven patients experienced multiple AEs, including transient hypotony, shallow anterior chamber, iris touch, and choroidal detachment [9]. The most common complications were transient hypotony (IOP < 5 mmHg after Day 1, which resolved by Day 90; 13%); shallow anterior chambers (13%), which occurred during the first 3 weeks after surgery; device touching the iris (13%); hyphema (9%); exposed Tenon’s capsule (9%); and transient choroidal detachment (9%) [9]. All complications were transient, occurring within the first 3 months after surgery, and resolved spontaneously [9]. No cases of erosion, device migration, leaks, infections, or persistent corneal edema were observed up to 3 years after implantation [9].

The MicroShunt implantation procedure was refined to provide less problematic blebs that are diffuse and posterior as a result of a wide and deep fornix-based subconjunctival/Tenon’s pocket and wide placement of MMC [9]. In Batlle et al. 2016, the typical bleb appearance tended toward shrinkage in volume and increased vascularity with time [9]. One case of an encysted bleb was observed, but controlled IOP was achieved after bleb revision [9]. One patient underwent reoperation with a second MicroShunt at 27 months due to an encapsulated bleb; the first device remained in place [9]. This patient’s treatment was considered a failure; however, the data were not excluded from the study [9].

### Ongoing studies of the MicroShunt

The MicroShunt is being investigated across the disease spectrum in the USA, Europe, Canada, Singapore, and Japan, with its effects being evaluated in patients with mild, moderate, and severe open-angle glaucoma [10–13, 29]. Results from recently completed studies with the MicroShunt are forthcoming. Furthermore, a large, pivotal randomized study is currently being conducted in 29 centers to evaluate its safety and effectiveness versus that of the gold standard, trabeculectomy (with adjunctive use of low-dose MMC [0.2 mg/mL for 2 min] for both procedures) [13]. Clinical follow up is scheduled over the course of the 2-year study [13]. Findings from this large study will aim to update the real-world practice in surgical management of patients with advanced progressive glaucoma.

## Conclusions

Suboptimal adherence to pharmacologic therapies [2] and substantial intra- and post-operative management associated with existing surgical approaches to glaucoma treatment [1, 3, 5, 30] highlight an unmet need in advanced and refractory glaucoma. Uncontrolled moderate-to-severe glaucoma is usually treated by trabeculectomy and/or large drainage valved/non-valved tube shunts [1]. These procedures are traumatic to the eye and, as a result, are delayed in the treatment paradigm until there are no remaining pharmacologic or surgical alternatives that can limit loss of visual function. The MicroShunt is a minimally invasive device that has the potential to be less traumatic to the eye than trabeculectomy and large drainage tube shunts; as such, MicroShunt surgery may be recommended earlier in the treatment paradigm before the optic nerve is severely damaged. This article reviews the early development and first clinical trials of the MicroShunt and demonstrates that a plateless tube made from SIBS (a unique biocompatible, bioinert biomaterial) can remain patent in the eye to address this unmet need. Considering the unique material and design, minimally invasive approach to implantation, and promising efficacy and safety profile demonstrated in the study described above, the MicroShunt may offer a solution to this gap in the glaucoma treatment armamentarium.

## Data Availability

Data sharing is not applicable to this article as no datasets were generated or analyzed during the current study.

## Abbreviations

AE: Adverse event
BL: Baseline
CE: Conformité Européenne
FDA: Food and Drug Administration
GLP: Good laboratory practice
IDE: Investigational device exemption
IOP: Intraocular pressure
MIDI: Miami InnFocus drainage implant
MIGS: Micro-invasive glaucoma surgery
MMC: Mitomycin C
OBC: Ophthalmic Biophysics Center
SD: Standard deviation
SIBS: Poly (styrene-block-isobutylene-block-styrene)
